# Supplementary material for: Lipid Droplets Are a Physiological Nucleoporin Reservoir
Source: Cells. 2021 Feb 22;10(2):472. doi: 10.3390/cells10020472 (PMC7926788; doi:10.3390/cells10020472)
Supplement: Supplementary file 1 [file cells-10-00472-s001.zip › Supplementary Files/Supplementary Figures Kumanski et al.pdf]

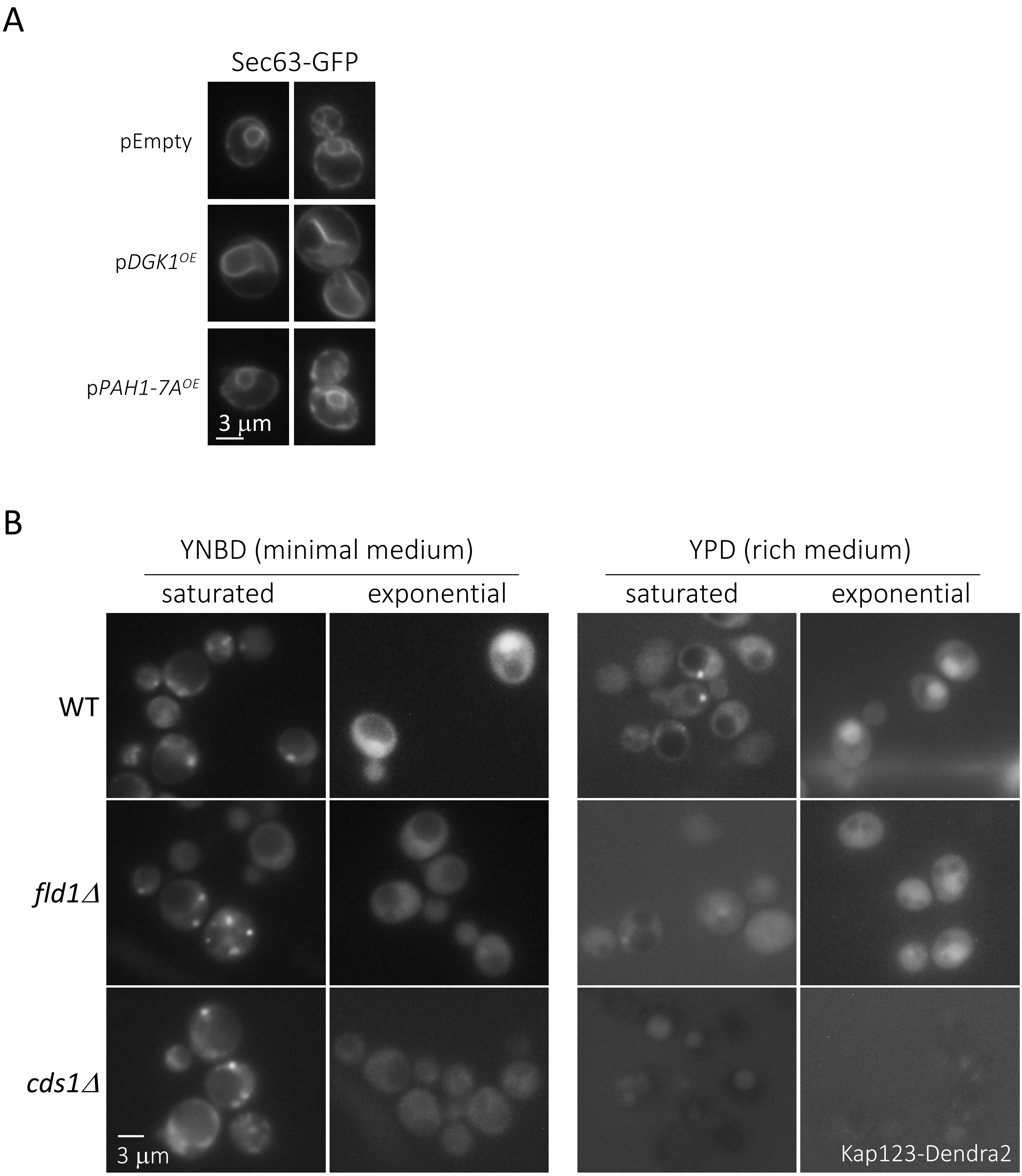


**Supplementary Figure 1. Additional information for Figures 4 and 6.**
**(A)** The same cells described in Figure 4 (transformed either with an empty plasmid, or with a plasmid overexpressing Dgk1 or *Pah1-7A*) were simultaneously transformed with another vector expressing a C-terminally GFP-tagged version of the ER marker Sec63. Observation of ER morphology permits the verification of the effect of the overexpression of Dgk1 and Pah1-7A, as described (Han G-S, O'Hara L, Siniossoglou S. Carman G, (2008) J Biol Cell; Karanasios E, Barbosa A.D., Sembongi H, Mari M, Han G.S., Reggiori F., Carman G.M., Siniossoglou S. (2013) Mol. Biol. Cell).
**(B)** Fluorescence microscopy images of WT and mutant cells in which Kap123 has been tagged with Dendra2. Cells were grown either in minimal, defined medium (YNBD) or rich medium (YPD), and images acquired either during exponential phase or after saturation.

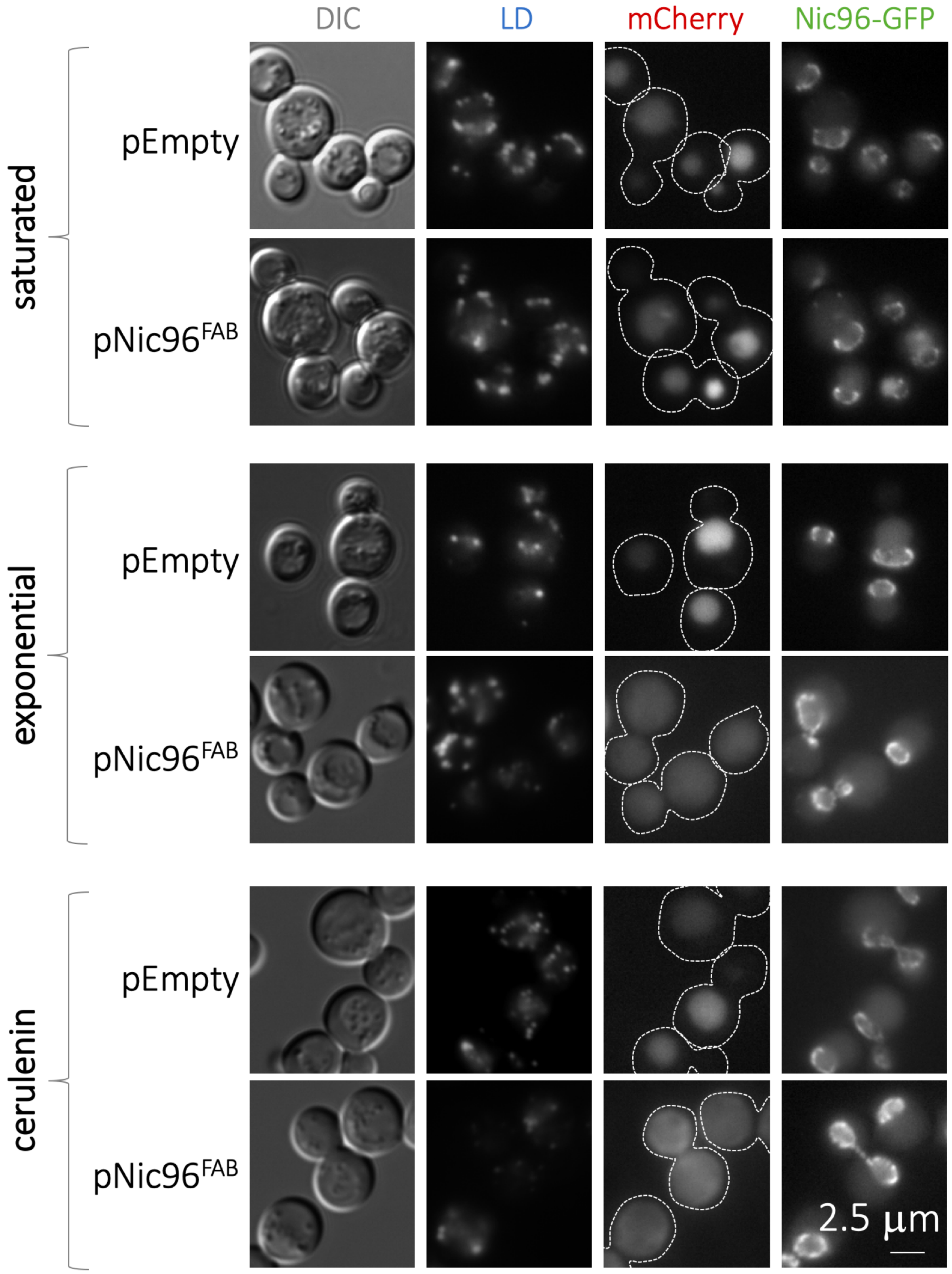


**Supplementary Figure 2. Nic96$^{FAB}$-mCherry diffuses in the cytoplasm during lipolysis.** Cultures of the otherwise WT Nic96-GFP strain were grown to saturation in minimal medium without uracil to select either for the vector expressing mCherry only (pEmpty) or Nic96$^{FAB}$-mCherry (pNic96$^{FAB}$). Then, cultures were split and either left untreated, or diluted for 3h, or diluted for 3h in the additional presence of 10 µg/mL cerulenin (named « saturated », « exponential » or « cerulenin », respectively). After that time, pictures were taken to evaluate the status of LD, the mCherry signal and the Nic96-GFP signals. The images are representative of the data obtained with four independent transformants of each construct. The contour of the individual cells, as determined by the DIC channel, has been over-imposed on the mCherry channel to clearly identify whether the signal is confined to one specific compartment, presumably the vacuole, or diffusing throughout the full cell.
